# Assessing the reliability of ethnicity data recorded in health-related administrative datasets in England.

**DOI:** 10.23889/ijpds.v7i3.1919

**Published:** 2022-08-25

**Authors:** Isobel Ward, Cameron Razieh, Rose Drummond, Vahé Nafilyan, Neil Bannister, Myer Glickman

**Affiliations:** 1 Office for National Statistics

## Objectives

During the COVID-19 pandemic, higher mortality among some ethnic minority groups was identified and has become the subject of significant public and government interest, highlighting an urgent requirement to quantify the reliability of ethnicity classification across health administrative data sets, which are utilised in health analysis and pandemic planning.

## Approach

The aim of our work was to assess how ethnicity data recorded in Census and health admin records varied across ethnicities and provide recommendations for how missingness can be accounted for by statisticians. Combining population level data from general practice (GP) records with hospital episode statistics (HES) for patients in England, we created a linked data set with Census 2011 data to reliably assess coverage and missingness between data sources. Most recent and modal ethnicity classifications were derived on a person-level from both HES and GP administrative data for comparison to gold-standard Census 2011 records.

## Results

Agreement rates were calculated to assess the reliability of ethnicity data recorded in health administrative datasets compared to Census data. We found that the agreement rates vary by ethnic group and other demographic characteristics. Furthermore, we highlighted groups of people who exist in one health-admin source, but not Census, and vice versa, illustrating the importance of accounting for the sample bias in health analysis when relying solely on primary or secondary care data sources. Implementation of techniques to account for bias and missingness were tested to propose methodology to improve reliability of ethnicity estimates from both HES and GP data, in order to ensure estimates of health disparities are as accurate as possible.

## Conclusion

We have linked GP records to Census 2011 and HES data to provide population-based ethnicity estimates of coverage, missingness and bias between data sources, in order to improve our understanding of ethnicity data quality. This work aims to inform policies tackling ethnic health inequalities in England.

